# What triggered narcolepsy: H1N1 vaccination, virus, or both? Important lessons learned from China

**DOI:** 10.1093/sleep/zsad005

**Published:** 2023-01-11

**Authors:** Jari K Gool, Mink S Schinkelshoek, Rolf Fronczek

**Affiliations:** Department of Neurology, Leiden University Medical Centre, Leiden, The Netherlands; Sleep-Wakecentre SEIN, Heemstede, The Netherlands; Anatomy and Neurosciences, Amsterdam UMC location Vrije Universiteit Amsterdam, Amsterdam, The Netherlands; Department of Neurology, Leiden University Medical Centre, Leiden, The Netherlands; Sleep-Wakecentre SEIN, Heemstede, The Netherlands; Department of Neurology, Leiden University Medical Centre, Leiden, The Netherlands; Sleep-Wakecentre SEIN, Heemstede, The Netherlands

The influenza A virus subtype H1N1 pandemic surfaced in the first month of 2009. Subsequently, a rigorous vaccination campaign began. With this came the first reports of a clearly increased incidence of narcolepsy in Scandinavian children [1]. The H1N1 vaccine named Pandemrix was suggested to be the culprit. Not long after, however, research groups from countries with a low vaccination grade (e.g. China, the United States, Taiwan, and several other European countries) reported a more modest increase in narcolepsy incidence [2–5]. A possible role for the H1N1 virus itself was thus emphasized. Increased incidences were mainly reported in children, and to a lesser extent in adults.

More than 10 years later, Wang et al., now consolidate one major piece of this puzzle by collecting the incidence of narcolepsy on a large scale over a 20-year period in mainland China with data from multiple sleep centers [6]. The incidence of both narcolepsy types *before* the H1N1 pandemic was 0.8 per 100 000 person-years. This increased to 3.1 *during* the pandemic, remaining somewhat higher (1.0) *after* the pandemic. This remained true when only considering clearly defined type 1 narcolepsy (88% of the 2869 included cases), and when excluding cases that had received prior vaccination against H1N1. Of note, the vaccine used for this in mainland China was not Pandemrix and was also not adjuvanted. Interestingly, the patients *during* the pandemic were of younger age (5–9 years old) compared to *before* and *after* the pandemic. The efforts of Wang et al. clearly show that the chance for people to develop narcolepsy type 1 increases when the H1N1 flu virus is rampant and activating the immune system. This is independent of vaccination against H1N1. Yet, as is well established and now also again seen in the Chinese data, only occurs in people with the DQB1*06:02 HLA allele.

Is the autoimmune hypothesis of narcolepsy thus proven? Hypocretin-deficient narcolepsy type 1 is assumed to be caused by the autoimmune destruction of hypothalamic hypocretin neurons [7]. Because the hypocretin peptides resemble parts of the H1N1 virus, cross-reactivity has been suggested [8]. Note that this theory does not explain narcolepsy type 2, in which hypocretin is not absent. People with this form of narcolepsy are thus mostly left out of studies involving the autoimmune hypothesis.

In 2018, hypocretin-specific T-cells were identified in the blood of people with narcolepsy type 1 [9]. This was a fascinating and crucial finding. However, these cells were primarily restricted by HLA-DR and not by HLA DQB1*06:02. Furthermore, there was no cross-reactivity with influenza peptides. Finally, similar T-cells were also found in a low percentage of healthy controls. The question thus remains if these T-cells truly reflect the primary narcolepsy disease mechanism. They might also represent secondary effects of hypocretin neurons damaged by an—as of yet—unknown other processes. Therefore, the exact role of HLA-DQB1*06:02 and autoreactive T-cells is still a mystery.

Is H1N1 still triggering narcolepsy across the globe? Since the 1918 Spanish flu pandemic, there has been almost a century in which the H1N1 virus has hardly been detected. The 2009–2010 pandemic changed this [10]. Yet, hypocretin-deficient narcolepsy also existed in the last century. This implies that (vaccination against) H1N1 is not the sole trigger. Multiple other candidates have been suggested. Most convincingly, streptococcal infections have been proposed [11, 12]. Whether there could be a role for non-H1N1 flu strains and other vaccinations in the development of narcolepsy has not been systematically studied.

Yet, persistent circulation of the H1N1 virus most likely still contributes to the development of new narcolepsy cases. A study involving 22 sleep centers across the US reported a 1.6-fold increase in pediatric cases after the 2009 pandemic [4]. Within Europe, a new child-specific narcolepsy type 1 incidence peak was seen in 2013 in the Netherlands, Italy, and France [13]. Of note, the 2010 narcolepsy peak was relatively mild in these countries. This may suggest the existence of a “limited pool” of people susceptible to developing narcolepsy. Wang et al. have also reported varying narcolepsy incidence rates in mainland China after 2010, with other peaks in 2012, and 2014. They have deemed the “limited pool” hypothesis unlikely since Chinese post-pandemic narcolepsy incidence rates did not normalize and generally remained increased.

Narcolepsy incidence generally peaked in spring in both mainland China and the United States, 4–8 months after the preceding H1N1 flu season intensity peak [4–6]. In contrast, two American cases have previously been identified in which narcolepsy symptoms directly started after H1N1 infection [4]. This would suggest that the presumed autoimmune process underlying narcolepsy could take between days–months to complete. In a large study in the United Kingdom [14], people were identified who had developed narcolepsy symptoms up to 5 years after their Pandemrix vaccination. In people with a quick onset of narcolepsy symptoms after H1N1 flu infection or vaccination, a causal relationship seems plausible. However, the possibility of long delays between possible immunological triggers and the development of narcolepsy remains to be shown.

To conclude, Wang et al. again show a clear relation between infection with the H1N1 flu virus and a relatively swift and severe development of narcolepsy type 1 in people with the DQB1*06:02 HLA allele. On top of this, data from Europe shows the adjuvanted H1N1 vaccine Pandemrix to play a role [2] and other infections (e.g. streptococcus) have also been implicated [11, 12]. This does not seem to leave a lot of room for doubt regarding the autoimmune nature of narcolepsy type 1. It is thus surprising that direct evidence for the suspected autoimmune process is still lacking. More than 20 years after the discovery of hypocretin deficiency in narcolepsy with cataplexy the ultimate question remains unsolved. What happens to the hypothalamic hypocretin neurons in narcolepsy type 1? Are the hypocretin neurons of genetically susceptible people indeed unrecoverably killed by T-cells that wrongly elicit an immune response to a self-antigen in hypocretin neurons after an immune trigger (or several triggers)? Or is the immune mechanism in reality aimed at another target outside hypocretin neurons? Are the hypocretin neurons really gone?
